# Construction and assessment of the quality of a care protocol for insulin use in hospitalized children and adolescents

**DOI:** 10.1590/0034-7167-2024-0098

**Published:** 2025-03-10

**Authors:** Sarah Arrais de Lavor, Carla Cristina de Sordi, Vanessa de Araújo Lima Freire, Débora Lira Correia, Rhanna Emanuela Fontenele Lima de Carvalho, Fernanda Jorge Magalhães, Sherida Karanini Paz de Oliveira

**Affiliations:** I Universidade Estadual do Ceará. Fortaleza, Ceará, Brazil

**Keywords:** Child, Adolescent, Insulin, Patient Safety, Hospitalization, Niño, Adolescente, Insulina, Seguridad del Paciente, Hospitalización

## Abstract

**Objectives:**

to develop and assess the quality of a care protocol for safe insulin use in hospitalized children and adolescents.

**Methods:**

a methodological study developed in three stages: scoping review, protocol development, and quality assessment. The scoping review followed the JBI recommendations, using the PRISMA-ScR checklist. The protocol was constructed according to the Guide for the Construction of Healthcare Protocols, and quality assessment was carried out byjudges using AGREE II.

**Results:**

the protocol achieved a quality rating of 94.4% in the scope and purpose domain; 90.4% in stakeholder involvement; 96.1% in rigor of development; 95.2% in clarity of presentation; 92.8% in applicability; 97.6% in editorial independence; and 92.8% for overall guideline assessment. Judges made suggestions, most of which were accepted.

**Final Considerations:**

the protocol was constructed and assessed as a quality instrument recommended for use by healthcare professionals in the context of insulin therapy in pediatrics.

## RESUMEN

**Objetivos:**

construir y evaluar la calidad de un protocolo de atención para el uso seguro de insulina en niños y adolescentes hospitalizados.

**Métodos:**

estudio metodológico, desarrollado en tres etapas: revisión del alcance, construcción del protocolo y evaluación de la calidad. La revisión del alcance siguió las recomendaciones del JBI, utilizando la lista de verificación PRISMA-ScR. La construcción del protocolo siguió la Guía para la Construcción de Protocolos de Atención y la evaluación de la calidad fue realizada por jueces mediante AGREE II.

**Resultados:**

el protocolo alcanzó una calidad del 94,4% en el dominio alcance y propósito; 90,4% en desarrollo de los interesados; 96,1% en rigor de desarrollo; 95,2% en claridad de presentación; 92,8% en aplicabilidad; 97,6% en independencia editorial; y 92,8% en la evaluación general de la guía. Los jueces hicieron sugerencias, la mayoría de las cuales fueron aceptadas.

**Consideraciones Finales:**

el protocolo fue construído y evaluado como un instrumento de calidad recomendado para su uso por profesionales de la salud en el contexto de la insulinoterapia en pediatría.

## INTRODUCTION

Insulin plays an essential role in blood glucose control^(1)^ and is classified as a high-alert medication (HAM) due to its significant participation in drug-related incidents. This categorization is attributed to the complexity of dosage and the diversity of pharmaceutical presentations available on the market^(2)^.

In hospital institutions, drug-related incidents are common, especially in the pediatric population, which is more vulnerable due to its specific characteristics, reflected in higher rates compared to adults^(3)^.

Medication errors in pediatrics can have serious implications for quality of care, resulting in possible irreversible harm to patients. These errors can arise in a variety of ways, such as excessive or insufficient doses, inappropriate route of administration, and administration of the correct medication to the wrong patient. Therefore, it is imperative to cultivate a culture of risk prevention by implementing reporting systems, training professionals, and standardizing procedures^(4)^.

A prevalence analysis of diabetes management in patients admitted to a metropolitan hospital in Melbourne identified the occurrence of diabetes-related medication errors in 20% (21/105) of hospitalized patients, in addition to significant therapeutic failure in the face of glycemic challenges. It is noteworthy that, of the 105 patients who were already being treated with hypoglycemic medications or insulin before admission, 21 presented 27 medication errors, 18 related to hypoglycemic medications and nine to insulin^(5)^.

In this regard, care protocols, continuing education and the adoption of double-checking procedures are recommended strategies to minimize errors in the use of insulin in healthcare settings^(6)^.

The care protocol is a tool for safe practice in relation to glycemic control, and, to achieve its ideal application, it is essential that the team fully understands this instrument, is confident in its handling and carrying out procedures, in order to offer safe and effective care^(7)^.

Given the above, the following questions arose: what content is necessary to develop a care protocol for the safe use of insulin in hospitalized children and adolescents? Does the care protocol for the safe use of insulin in hospitalized children and adolescents have evidence of content validity?

Thus, the scientific and social relevance of this research is to provide technology for the care of hospitalized children and adolescents based on scientific evidence and validated by experts as well as to ensure standardization of conduct, better interaction between interdisciplinary teams, optimization of care in decisionmaking and consequently quality in the care provided to this public.

## OBJECTIVES

To develop and assess the quality of a care protocol for the safe use of insulin in hospitalized children and adolescents

## METHODS

### Ethical and legal aspects

The research met the ethical and legal requirements of Resolution 466/2012 of the Brazilian National Health Council (CNS – *Conselho Nacional de Saúde*), and was approved by the *Hospital Infantil Albert Sabin* (CE) Research Ethics Committee, whose opinion is attached to the submission. All participants signed the Informed Consent Form (ICF).

### Study design and period

This is a methodological study, carried out from April to December 2023, for developing a care protocol for the safe use of insulin in hospitalized children and adolescents, divided into three stages: 1) scoping review on the topic; 2) care protocol construction aimed at the use of health professionals regarding insulin therapy in children and adolescents; 3) care protocol quality assessment by expert judges.

To provide a solid basis for transparent and complete study assessment, the Appraisal of Guidelines Research & Evaluation II (AGREE II) checklist, a tool for improving clinical practice guideline reporting available on the EQUATOR platform, was used.

### Population and sample

The search for judges in the area of interest of the study (insulin therapy, pediatrics and patient safety) was carried out on the *Lattes* Platform, and snowball sampling was used. To be selected, the judges had to meet at least two of the criteria stipulated by Jasper^(8)^, namely: having skills/knowledge acquired through experience; having specialized skills/knowledge that make professionals an authority on the subject; having special skills in a certain study design; and having a high rating given by an authority

Seven judges were identified and invited by means of an invitation letter, highlighting the reason for choosing them as experts, the study objectives, instrument description and its scoring and interpretation, in addition to an explanation of the response form, conceptual definitions that gave rise to the instrument, the dimensions involved and the measurement model used to provide the instrument’s conceptual and theoretical bases^(9)^. Upon acceptance, the ICF, a copy of the care protocol and the data collection instrument were sent via an online form.

A period of four weeks was granted for material assessment and return. In the second week, a new contact was made, informing the researcher of the availability to clarify doubts and provide information about the instrument. At the end of the period, feedback was obtained from all seven judges who made up the sample of this research.

### Study protocol

#### Stage I – Scoping review

The scoping review followed five phases, such as research question identification, relevant study identification, study selection, data mapping, and grouping, synthesis and reporting of results^(10)^, prepared using recommendations from the Preferred Reporting Items for Systematic Reviews and Meta Analyses - extension for Scoping Reviews (PRISMA-ScR) checklist^(11)^.

To define the research question, the PCC strategy was used, an acronym for Population, Concept and Context^(12)^, structured in P: hospitalized children and adolescents, C: safe practices and C: insulin therapy, culminating in the guiding question: what are the good practices related to insulin therapy in hospitalized children and adolescents?

Studies on safe insulin therapy practices in hospitalized children and adolescents aged 0 to 18 years, available in full text and free of charge and without a time frame, were included. All selected records were exported to Rayyan (Qatar Computing Research Institute) for automatic removal and exclusion of duplicates^(13)^.

The search strategy was drawn up based on the Health Sciences Descriptors (DeCS)/Medical Subject Headings (MeSH) and Emtree (EMBASE), together with the Boolean operators AND and OR.

The search equation was adapted to each database, with the aim of identifying the one that was most sensitive for selecting studies relevant to the research. Some articles were also included through the recovery of the references found, in addition to the Brazilian Diabetes Society’s search engine^(1)^.

Data collection took place in the second half of 2023 in the Medical Literature Analysis and Retrieval System Online (MEDLINE) (n=561), Scopus (n=299), Web of Science (n=202), Cumulative Index to Nursing & Allied Health Literature (CINAHL) (n=118), Latin American and Caribbean Literature in Health Sciences (LILACS) (n=126), Cochrane Library (n=56) and Embase (n=398) databases, totaling 1,760 articles.

Article search and selection were carried out by two reviewers, independently, in order to ensure greater methodological rigor, and any disagreements were resolved at the time of detection, so as not to compromise the methodological progress. The procedure followed by reading titles, abstracts and, later, full articles for analysis, observing whether they addressed the study guiding question.

Sequentially, a data extraction instrument was completed based on the JBI model^(12)^, including article code, authorship, year of publication, country, language, methodological design, good practices and main recommendations.

Article interpretation and discussion were carried out according to their categories and analyses. Some articles that best fit the criteria and contributed to the understanding and resolution of the guiding question were listed. Therefore, a careful review of all selected articles was carried out, which allowed the analysis of the information and synthesis of existing knowledge.

In this review, to process the results, studies were qualitatively summarized and analyzed descriptively, with a synthesis of each study included in the sample. Forthe narrative synthesis of evidence, the results were grouped and presented in charts.

#### Stage II - Protocol construction

To guide the protocol development, criteria for assessing and constructing care/assistance protocols described by Pimenta^(14)^ were adopted in the following stages: origin; objective; development group; conflict of interest; evidence; review; flowchart; outcome indicator; protocol quality assessment (judges assessed the protocol using the instrument for assessing clinical guidelines-AGREE II^(15)^).

For the implementation plan, training was planned to present the protocol and raise awareness among the team about its use. In addition to this, the protocol can also be disseminated on the intranet, scientific journals and health events.

#### Stage III - Quality assessment by judges

The data collection instrument consisted of two parts: sociodemographic and professional data and the instrument for protocol assessment (AGREE II). The first instrument for characterizing judges presented variables on sex, age, state of origin, area of training, time since training, qualification, and time of experience with insulin therapy in hospitalized children.

AGREE II has 23 items, distributed across six domains: scope and purpose; stakeholder involvement; rigor of development; clarity of presentation; applicability; and editorial independence^(15)^.

The overall guideline assessment requires AGREE II users to make a judgment about the quality of the guideline, taking into account the assessment items considered in the process. It occurs after assessing the 21 items and is proposed by a Likert-type scale, ranging from 1 to 7, with a score of 1 meaning the lowest possible quality and a score of 7 the highest possible quality. After this assessment, there must be an opinion recommending the guideline for use in clinical practice^(15)^.

AGREE-II does not define a cut-off point for classifying quality. This decision should be made by the author and guided by the context in which the protocol is used^(15)^. At the end of assessment, judges classified and assessed the protocol as follows: would recommend; would recommend with limitations; and would not recommend.

## RESULTS

This technology was entitled “Care protocol for safe use of insulin in hospitalized children and adolescents”. In the first stage, 1,760 articles were identified in the databases, and 489 were excluded because they were duplicates. A total of 1,271 articles were eligible for title and abstract reading. After exclusion, 25 were assessed in full, and 13 did not answer the research question. Therefore, 12 articles were selected. However, after in-depth reading and analysis of the references, four articles were recovered, totaling 16 articles.

The studies were divided into categories: appropriate technique for preparing subcutaneous insulin injections (seven articles); use of the skinfold technique (eight articles); skin inspection and choice of injection site (five articles); use of appropriate devices (ten articles); intravenous insulin infusion (five articles); prevention of complications and management of pain during injection (12 articles); blood glucose monitoring and hospital hypoglycemia/ hyperglycemia patterns (four articles); insulin storage and conservation (seven articles); and correct disposal of sharps (seven articles). These categories determined the protocol structure in nine topics with the same name.

Each topic contains information about the objective, description of care and observations, which were organized according to scientific evidence found in the scoping review. The title of each topic was highlighted in orange, which is related to patient safety, for better organization and visibility.

Seven judges participated in the content validity process, all women and nurses, with an average age of 44+7.9 years and the majority from the state of São Paulo (5), followed by Ceará (2). All are specialists, four with master’s degrees and one with a doctoral degree, and have an average of 11 +8.4 years of clinical experience with hospitalized children and adolescents using insulin.

In the assessment process, scores were achieved according to each domain of the instrument (Chart 1).

**Chart 1:** Distribution of scores and protocol suitability according to the Appraisal of Guidelines for Research & Evaluation II domains, Fortaleza, Ceará, Brazil, 2024

**Domain 1 – Scope and purpose**	**J1**	**J2**	**J3**	**J4**	**J5**	**J6**	**J7**	**TOTAL**
1. The guideline general objectives are specifically described.	7	7	7	7	6	6	7	47
2. The health issues covered by the guideline are specifically described.	7	7	7	7	5	6	7	46
3. The population (patients, public, etc.) for whom the guideline is intended is specifically described.	7	7	7	7	6	6	7	47
Total	21	21	21	21	17	18	21	140
Suitability for the domain 1 – 94.4%								
Domain 2 – Stakeholder involvement	**J1**	**J2**	**J3**	**J4**	**J65**	**J6**	**J7**	**TOTAL**
4. The guideline development team includes ndividuals from all relevant professional groups.	7	7	7	7	5	7	6	46
5. We sought to understand the target population’s opinions and preferences (patients, public, etc.).	7	1	7	7	7	6	6	41
6. The guideline target users (patients, public, etc.) are clearly defined.	7	7	7	7	7	6	7	48
Total	21	21	21	21	19	19	19	135
Suitability for the domain 2 – 90.4%								
**Domain 3 – Rigor of development**	**J1**	**J2**	**J3**	**J4**	**J5**	**J6**	**J7**	**TOTAL**
7. Systematic methods were used to search for evidence.	7	7	7	7	7	6	7	48
8. Criteria for selecting evidence are clearly described.	7	7	7	7	7	6	7	48
9. Strengths and limitations of the body of evidence are clearly described.	7	7	7	7	6	6	7	47
10. Methods for formulating recommendations are clearly described.	7	7	7	7	5	6	7	46
11. The benefits, side effects and health risks were considered in formulating the recommendations.	7	7	7	7	6	6	7	47
12. There is an explicit link between the recommendations and the evidence supporting them.	7	7	7	7	6	6	7	47
13. The guideline was externally reviewed by experts before publication.	7	7	7	7	7	6	7	48
14. A procedure for updating the guideline is available.	7	7	7	7	7	6	7	48
Total	56	56	56	56	51	48	56	379
Suitability for the domain 3 – 96.1%								
**Domain 4 – Clarity of presentation**	**J1**	**J2**	**J3**	**J4**	**J5**	**J6**	**J7**	**TOTAL**
15. Recommendations are specific and unambiguous.	7	7	7	7	6	6	7	47
16. Different options for addressing the health condition or problem are clearly presented.	7	7	7	7	6	6	7	47
17. Key recommendations are easily identified.	7	7	6	7	7	6	7	47
Total	21	21	20	21	19	18	21	141
Suitability for the domain 4 – 95.2%								
**Domain 5 – Applicability**	**J1**	**J2**	**J3**	**J4**	**J5**	**J6**	**J7**	**TOTAL**
18. The guideline describes the facilitators and barriers to its implementation.	7	7	7	7	6	6	7	47
19. The guideline provides advice and/or tools on how the recommendations can be put into practice.	7	7	7	7	6	6	7	47
20. The potential resource implications arising from the implementation of recommendations have been considered.	7	7	7	7	6	6	6	46
21. The guideline presents criteria for its monitoring and/or auditing.	7	4	7	7	6	6	7	44
Total	28	25	28	28	24	24	27	184
Suitability for the domain 5 – 92.8%								
**Domain 6 – Editorial independence**	**J1**	**J2**	**J3**	**J4**	**J5**	**J6**	**J7**	**TOTAL**
22. The opinion of the funding body did not influence the guideline content.	7	7	7	7	7	6	7	48
23. Conflicts of interest of team members who developed the guideline were recorded and addressed.	7	7	7	7	7	6	7	48
Total	14	14	14	14	14	12	14	96
Suitability for the domain 6 – 97.6%								

It can be seen that domain 3 (rigor of development) and domain 4 (clarity of presentation) obtained the best scores. Domain 2 (stakeholder involvement) obtained the lowest score. However, it can be seen that all domains exceeded the minimum value of 80% defined as “satisfactory quality” and achieved rates above 90%.

Regarding the overall protocol assessment and the recommendation according to judges, we highlight that four gave a score of 7 and three gave a score of 6, with the total percentage of the overall protocol assessment being equal to 92.8%. Therefore, it is considered that the protocol is of high quality. Furthermore, six judges recommended using the protocol without restriction, and only one recommended it with modifications.

Judges made suggestions regarding the instrument content for the protocol items and domains, according to AGREE II (Chart 2), which, for the most part, were accepted.

**Chart 2:** Judges’ suggestions and comments regarding the protocol content, Fortaleza, Ceará, Brazil, 2023

Judge	Suggestion	Decision-making
Judge 1	Describe the insulin application sites better with illustrations.	Accepted and included illustration with application locations.
Judge 2	I believe that an interesting indicator for monitoring/auditing the post-implementation protocol is the presence of complications related to the insulin application technique.	Accepted.
Judge 3	I suggest modifying the topic that contains the appropriate technique for administering subcutaneous insulin: in a hospital environment, skin antisepsis is performed in a unilateral movement with two swabs of 70% alcohol or cotton soaked in 70% alcohol. Circular movements are no longer used.	Topic III, in description of care Change to: in a hospital environment, skin antisepsis should be performed with unilateral movements using 70% alcohol swabs or cotton soaked in 70% alcohol.
Judge 4	1. Include the order of air injection along with the observation of aspiration of NPH and regular insulins to facilitate handling; 2. I was unsure about the sentence that says in the observation that a pen is safer for health professionals, since most places do not use the safety needle for the pen, making it necessary to disconnect it, which creates more risk than the syringe; 3. I would reassess the following sentence in the text, perhaps including in the observation: 45-degree angle on the 6 mm needle and the order of absorption.	Accepted.
Judge 5	I suggest including in more detail the step-by-step process for administering insulin in pens as well as including the use of technologies related to safe practices in insulin therapy (i-port, CGM, insulin pump, etc.).	The suggestion to include the step-by-step guide for administering insulin using pens was accepted. However, it was not possible to include the technologies as they were not the focus of the protocol.
Judge 7	Regarding this topic (target audience), I believe it would be beneficial to the psychology professional involved. When dealing with children, the challenge is to ensure acceptance of the technique and adequate rotation.	As this is a protocol for preparing and administering insulin, it is believed that, as it is a technical procedure, the psychologist would not benefit from this protocol.

Furthermore, in addition to suggestions, judges also made other comments and compliments, as stated below:

*Congratulations on your work!* (J1)

*I really liked the work done, very well written, easy to understand and of great value for clinical practice!!!* (J2)

*Congratulations on the work done.* (J5)

*A very dynamic training strategy is needed to achieve all these points. It is a long training; I suggest breaking it down into categories lasting no more than 30 minutes for better absorption and understanding by the team. Congratulations on the project.* (J7)

## DISCUSSION

The construction stage of this technology, with content mapped and synthesized from the review, supported the theoretical part, addressing the main practices for the safe and effective use of insulin in hospitalized children and adolescents. The protocol constructed aims to offer recommendations based on scientific evidence to health professionals, especially the nursing team, who work in the care of hospitalized children and adolescents using insulin therapy.

Insulin treatment is a complex process, and the need for this hormone in children and adolescents is dynamic, due to the physiological and behavioral changes characteristic of this age group, as well as patterns of daily routines, presence of concomitant diseases, among other factors^(16)^. In this sense, technologies, when applied and tested in hospital settings, present satisfactory results in care processes, since they facilitate the dynamics of professionals’ service, reduce the possibility of adverse events arising from the care provided and contribute to patient safety^(17)^.

The study carried out by Poppy *et al*.^(18)^ showed that the rate of hypoglycemic events in hospitalized children using insulin reduced from 1.4 preventable events per 100 days of insulin to 0.4, a reduction of 28% after the hospital unit established multiple interventions, such as development of protocols, continuing education of professionals on insulin therapy and real-time monitoring of patients at risk of unexpected hypoglycemia.

The care provided to children in hospitalization is becoming increasingly improved, as the search for improvements in the instruments used in daily care, scientifically based on methods that make them valid, has been a crucial point developed by nurses who work with the aim of transforming practice in the face of methodological innovations^(19)^.

Care technologies developed based on robust evidence and assessed by experts in the field contribute positively to care, given that they combine science and the daily experience of professionals^(20)^.

Other studies related to the theme of this study have also used AGREE II, such as the one carried out in Palestine, which aimed to assess a clinical guideline for diabetes mellitus. And it presented lower rates than those found in our study, with a low quality assessment: domain 1 (66.7%); domain 2 (35%); domain 3 (36.5%); domain 4 (61.5%); domain 5 (27%) and domain 6 (40%). The use was recommended with modifications by 12 evaluators and not recommended by four evaluators^(21)^.

Some guidelines for safe practices related to insulin use in hospitalized children and adolescents are: skinfolds are mandatory in children under 6 years of age and in thin adolescents; permanently attached needle syringes offer better dose accuracy and reduced dead space; short needles (4, 5 and 6 mm in length) are ideal for all patients, and their use is safe for any individual; the absorption of insulin injected into sites with lipohypertrophy is unpredictable, and there may be greater glycemic variability^(22)^.

Some care must be taken when preparing insulin: cloudy or premixed insulin must be resuspended before each injection; an air injection of two units must be made before preparing the intended dose to ensure free and unobstructed flow into the pen needle^(23)^.

Insulin must be administered subcutaneously to ensure optimal absorption. It is important to inspect the skin before and after injection, insert the needle correctly into the subcutaneous tissue, leave the needle in the tissue long enough for the entire dose of insulin to be absorbed, and rotate injection sites. These actions contribute to efficient metabolic control^(24)^.

The use of short, thin needles (4 mm and 5 mm) in insulin administration improves injection technique by decreasing pain during the injection process, reducing the chance of intramuscular injection, and improving the rate and completeness of insulin absorption^(25)^.

Injection using a vial and syringe is considered an effective and cheaper way of administering insulin. However, it has some drawbacks, such as low dose accuracy, lackof social acceptance, fear, and difficulty in transporting. Insulin pens, on the other hand, provide more flexibility, precision, and discretion compared to syringes, contributing to improved adherence to treatment^(26)^.

Proper storage and preservation are essential to ensure insulin efficacy and successful therapy. Insulin in use can be kept at room temperature (below 30°C) for up to 30 days. It is important to note that administering refrigerated insulin may be uncomfortable for patients^(23)^.

Finally, safe disposal of used sharps should be practiced in both home and hospital settings to prevent needlestick injuries related to insulin needles^(27)^.

Local complications of insulin therapy are common in patients with diabetes, such as infections and lipodystrophy, the latter having the greatest impact on metabolic control. A study conducted in Turkey in children with type 1 diabetes found this complication in 11.8% of patients. Patients with lipodystrophy used higher median doses of insulin (0.97 U/kg/day) than those who did not use it (0.78 U/kg/day; p = 0.016), and had frequent hypoglycemia (p = 0.007)^(28)^.

Incorrect rotation of insulin injection sites and reuse of the needle more than five times are significantly associated with the development of lipodystrophy, thus highlighting the importance of correct injection technique^(29)^.

In hospitalized individuals with diabetes, it is necessary to monitor blood glucose levels according to their clinical condition. For those who are eating, monitoring should be performed before meals; for those on a zero-calorie diet, it is recommended to check capillary blood glucose levels every four to six hours. More frequent monitoring of blood glucose levels (30 minutes to 2 hours) is necessary for the safe use of intravenous insulin therapy. It is important to note that if the glucose result does not correlate with patients’ clinical condition, it should be confirmed by measuring a laboratory sample^(30)^.

It is confirmed that the effectiveness of insulin therapy depends on the correct use of the injection technique. However, despite evidence that an effective technique is essential to minimize errors and improve glycemic control, there is still a need to raise awareness among patients and health professionals about the importance of optimizing insulin injection techniques^(25)^.

The implementation of insulin administration education programs in health care settings, such as hospitals, wards and Intensive Care Units, is essential to ensure the safe and effective use of insulin, establishing standard procedures, reducing the risk of inappropriate insulin prescription and administration and improving patient outcomes^(25,26,27)^.

These programs should address all aspects of safe and rational insulin use, in addition to indicators that can be used to quantify the success of the program, such as reduction of errors in insulin administration, reduction in hypoglycemia rates, percentage of correct management of hypoglycemia, percentage of patients and professionals who completed the insulin program, correct disposal of needles and syringes^(27)^, indicators similar to those in the protocol.

### Study limitations

The limitations of this study were determined by the lack of studies with high levels of evidence that elucidate specific good practices on insulin use aimed at the pediatric population. It is believed that future research on this topic is essential to improve knowledge and ensure qualified and safe care.

### Contributions to nursing

This protocol presents safe guidelines for the practice of insulin therapy in hospitalized children and adolescents, contributing significantly to the improvement of clinical nursing practice, promoting quality of care.

The use of this protocol will favor insulin therapy standardization, with a reduction in medication errors associated with insulin, especially in the pediatric setting, since it has particularities such as dosages and adverse reactions, a fact that may contribute to better clinical and metabolic results in children and adolescents.

## FINAL CONSIDERATIONS

The “Care protocol for safe use of insulin in hospitalized children and adolescents” was constructed with methodological rigor, assessed and recommended by expert judges for use by health professionals in the context of insulin therapy in pediatrics.

The product of this research may contribute to clinical nursing practice, in order to aggregate knowledge, standardize conduct and mitigate errors and/or complications related to the use of insulin in hospitalized children and adolescents.

It is suggested that further studies be carried out for implementation and monitoring as well as to assess the impact of the use of the protocol on the quality and safety of care for children and adolescents undergoing insulin therapy in the hospital setting, in addition to the periodic updating of the protocol.

## Data Availability

https://doi.org/10.17605/OSF.IO/JN6CQ
