# Supplementary material for: Construction and assessment of the quality of a care protocol for insulin use in hospitalized children and adolescents
Source: Rev Bras Enferm. 2025 Mar 10;78(1):e20240098. doi: 10.1590/0034-7167-2024-0098 (PMC11893052; doi:10.1590/0034-7167-2024-0098)
Supplement: Translation

# Translation: Construção e avaliação da qualidade de protocolo assistencial para uso de insulina em crianças e adolescentes hospitalizados

**DOI:** 10.1590/0034-7167-2024-0098pt

**Authors:** Sarah Arrais de Lavor, Carla Cristina de Sordi, Vanessa de Araújo Lima Freire, Débora Lira Correia, Rhanna Emanuela Fontenele Lima de Carvalho, Fernanda Jorge Magalhães, Sherida Karanini Paz de Oliveira

**Affiliations:** I Universidade Estadual do Ceará. Fortaleza, Ceará, Brasil

**Keywords:** Criança, Adolescente, Insulina, Segurança do Paciente, Hospitalização

## Abstract

**Objetivos:**

construir e avaliar a qualidade de um protocolo assistencial para uso seguro de insulina em crianças e adolescentes hospitalizados.

**Métodos:**

estudo metodológico, desenvolvido em três etapas: revisão de escopo, construção de protocolo e avaliação da qualidade. A revisão de escopo seguiu as recomendações da JBI, utilizando o *checklist* PRISMA-ScR. A construção do protocolo seguiu o Guia para a Construção de Protocolos Assistenciais, e a avaliação da qualidade foi realizada por juízes por meio do AGREE II.

**Resultados:**

o protocolo obteve qualidade de 94,4% no domínio escopo e finalidade; 90,4% no desenvolvimento das partes interessadas; 96,1 % no rigor do desenvolvimento; 95,2% na clareza da apresentação; 92,8% na aplicabilidade; 97,6% na independência editorial; e 92,8% na avaliação global. Os juízes fizeram sugestões, acatadas em sua maioria.

**Considerações Finais:**

o protocolo foi construído e avaliado como instrumento de qualidade recomendado para uso por profissionais de saúde no contexto da insulinoterapia em pediatria.

## INTRODUÇÃO

A insulina desempenha um papel essencial no controle da glicose sanguínea^(1)^, sendo classificada como medicamento de alta vigilância (MAV) devido à sua significativa participação em incidentes relacionados a medicamentos. Essa categorização é atribuída à complexidade na dosagem e à diversidade de apresentações farmacêuticas existentes no mercado^(2)^.

Nas instituições hospitalares, incidentes relacionados a medicamentos são comuns, com destaque para a população pediátrica, mais vulnerável devido às suas características específicas, refletidas em taxas mais elevadas em comparação com adultos^(3)^.

O erro de medicação em pediatria pode ter sérias implicações na qualidade do atendimento, resultando em possíveis danos irreversíveis aos pacientes. Esses erros podem surgir de várias maneiras, como doses excessivas ou insuficientes, escolha da via de administração inadequada e administração do medicamento correto a um paciente errado. Portanto, é imperativo cultivar uma cultura de prevenção de riscos, implementando sistemas de notificação, capacitando os profissionais e padronizando condutas^(4)^.

Uma análise de prevalência acerca do manejo do diabetes em pacientes internados em hospital metropolitano de Melbourne identificou a ocorrência de erros de medicação relacionados ao diabetes em 20% (21/105) dos pacientes hospitalizados, além de falha terapêutica significativa diante de desafios glicêmicos. Destaca-se que, dos 105 pacientes que já estavam em tratamento com medicamentos hipoglicemiantes ou insulina antes da internação, 21 apresentaram 27 erros de medicação, sendo 18 relacionados aos medicamentos hipoglicemiantes e nove à insulina^(5)^.

Nesse sentido, os protocolos assistenciais, a educação continuada e a adoção de procedimentos de dupla verificação são estratégias recomendadas para minimizar falhas na utilização de insulina em ambientes de saúde^(6)^.

O protocolo assistencial é uma ferramenta para uma prática segura em relação ao controle glicêmico, e, para atingir o ideal de sua aplicação, é fundamental que a equipe compreenda plenamente esse instrumento, tenha segurança em seu manuseio e realização de procedimentos, de modo a oferecer uma assistência segura e eficaz^(7)^.

Diante do exposto, surgiram os questionamentos: qual o conteúdo necessário para elaboração de um protocolo assistencial para uso seguro de insulina em crianças e adolescentes hospitalizados? O protocolo assistencial para uso seguro de insulina em crianças e adolescentes hospitalizados tem evidências de validade de conteúdo?

Dessa forma, a relevância científica e social desta pesquisa é disponibilizar uma tecnologia no cuidado a crianças e adolescentes hospitalizados baseada em evidências científicas e validada por *experts,* assim como garantir a padronização de condutas, uma melhor interação entre as equipes interdisciplinares, a otimização da assistência na tomada de decisões e consequentemente qualidade no atendimento realizado a esse público.

## OBJETIVOS

Construir e avaliar a qualidade de um protocolo assistencial para uso seguro de insulina em crianças e adolescentes hospitalizados

## MÉTODOS

### Aspectos éticos e legais

A pesquisa atendeu aos requisitos éticos e legais da Resolução n° 466/2012 do Conselho Nacional de Saúde (CNS), e foi aprovada pelo Comitê de Ética em Pesquisa do Hospital Infantil Albert Sabin (CE), cujo parecer está anexado à submissão. Todos os participantes assinaram o Termo de Consentimento Livre e Esclarecido (TCLE).

### Tipo e período de estudo

Trata-se de estudo metodológico, realizado de abril a dezembro de 2023, para o desenvolvimento de protocolo assistencial para o uso seguro de insulina em crianças e adolescentes hospitalizados, dividido em três etapas: 1) revisão de escopo sobre a temática; 2) construção do protocolo assistencial direcionado para o uso dos profissionais de saúde acerca da insulinoterapia em crianças e adolescentes; 3) avaliação da qualidade do protocolo assistencial por juízes especialistas.

Para fornecer uma base sólida para a avaliação transparente e completa do estudo, foi utilizado o *checklist Appraisal of Guidelines Research & Evaluation II* (AGREE II), uma ferramenta para melhorar os relatórios de diretrizes de prática clínica disponível na plataforma EQUATOR.

### População e amostra

Procedeu-se à busca dos juízes na área de interesse do estudo (insulinoterapia, pediatria e segurança do paciente) na Plataforma *Lattes* e amostragem por bola de neve. Para serem selecionados, os juízes tiveram que obedecer a pelo menos dois desses critérios estipulados por Jasper^(8)^, quais sejam: possuir habilidade/conhecimento adquiridos pela experiência; possuir habilidade/conhecimento especializado que torna o profissional uma autoridade no assunto; possuir habilidade especial em determinado tipo de estudo; e possuir classificação alta atribuída por uma autoridade

Foram identificados sete juízes, sendo convidados por meio de uma carta convite, salientando o motivo de escolha como especialista, os objetivos do estudo, a descrição do instrumento e a sua pontuação e interpretação, além de uma explicação sobre a forma de resposta, definições conceituais que deram origem ao instrumento, as dimensões envolvidas e o modelo de medida usados para fornecer as bases conceituais e teóricas do instrumento^(9)^. Após o aceite, foram enviados o TCLE, uma cópia do protocolo assistencial e o instrumento de coleta de dados via formulário *online.*

Concedeu-se um prazo de quatro semanas para avaliação e retorno do material. Na segunda semana, realizou-se novo contato, informando a disposição da pesquisadora para esclarecimentos de dúvidas e informações sobre o instrumento. Ao final do prazo, obteve-se retorno de todos os sete juízes que compuseram a amostra da presente pesquisa

### Protocolo do estudo

#### Etapa I - revisão de escopo

A revisão de escopo seguiu cinco fases, tais como identificação da questão de pesquisa, identificação dos estudos relevantes, seleção de estudos, mapeamento dos dados, e agrupamento, resumo e relato de resultados^(10)^, elaboradas mediante recomendações do *checklist Preferred Reporting Items for Systematic Reviews and Meta Analyses - extension for Scoping Reviews* (PRISMA-ScR)^(11)^.

Para definição da questão de pesquisa, utilizou-se a estratégia PCC, acrônimo de População, Conceito e Contexto^(12)^, estruturado em P: crianças e adolescentes hospitalizados, C: práticas seguras e C: insulinoterapia, culminando na questão norteadora: quais as boas práticas relacionadas à insulinoterapia em crianças e adolescentes hospitalizados?

Foram incluídos estudos acerca das ações de prática segura de insulinoterapia em crianças e adolescentes hospitalizados pertencentes à faixa etária de 0 a 18 anos, disponíveis em texto completo e gratuito e sem recorte temporal. Todos os registros selecionados foram exportados para o *software* Rayyan (*Qatar Computing Research Institute*) para remoção automática e exclusão dos duplicados^(13)^.

A estratégia de busca foi traçada a partir dos Descritores em Ciências da Saúde (DeCS)/*Medical Subject Headings* (MeSH) e Emtree (EMBASE), em conjunto com os operadores booleanos *AND* e *OR*.

A equação de busca foi adaptada a cada base, com o intuito de identificar aquela que fosse mais sensível para seleção dos estudos pertinentes à pesquisa. Também foram contemplados alguns artigos por meio da recuperação das referências encontradas, além da diretriz da Sociedade Brasileira de Diabetes^(1)^.

A coleta de dados ocorreu no segundo semestre de 2023 nas bases de dados *Medical Literature Analysis and Retrieval System Online* (MEDLINE) (n=561 ), Scopus (n=299), *Web of Science* (n=202), *Cumulative Index to Nursing & Allied Health Literature* (CINAHL) (n=118), Literatura Latino-Americana e do Caribe em Ciências da Saúde (LILACS) (n=126), *Cochrane Library* (n=56) e Embase (n=398), totalizando 1.760 artigos.

A busca e a seleção dos artigos foram realizadas por dois revisores, de forma independente, no intuito de conferir maior rigor metodológico, sendo as discordâncias solucionadas no devido instante da detecção, a fim de não comprometer o prosseguimento metodológico. Seguiu-se com o procedimento de leitura de títulos, resumos e, posteriormente, artigos completos para análise, observando se esses contemplavam a questão norteadora do estudo.

Sequencialmente, foi preenchido um instrumento para extração de dados com base no modelo do JBI^(12)^, contemplando código do artigo, autoria, ano de publicação, país, idioma, delineamento metodológico, boas práticas e principais recomendações.

A interpretação e a discussão dos artigos foram realizadas de acordo com suas categorias e análises. Foram elencados alguns artigos que mais se encaixaram nos critérios e que contribuíram para o entendimento e resolução da questão norteadora. Assim sendo, foi realizada revisão criteriosa de todos os artigos selecionados, o que permitiu a análise das informações e resumo do conhecimento existente.

Nesta revisão, para o tratamento dos resultados, os estudos foram sumarizados qualitativamente e analisados de forma descritiva, com a síntese de cada estudo incluído na amostra. Para a síntese narrativa das evidências, os resultados foram agrupados e apresentados em quadros.

#### Etapa II - Construção do protocolo

Para guiar a elaboração do protocolo, foram adotados critérios de avaliação e construção de protocolos de assistência/cuidados descritos por Pimenta^(14)^ nas seguintes etapas: origem; objetivo; grupo de desenvolvimento; conflito de interesses; evidências; revisão; fluxograma; indicador de resultado; avaliação da qualidade do protocolo (os juízes avaliaram o protocolo por meio do instrumento para avaliação de diretrizes clínicas - AGREE II^(15)^).

Para o plano de implementações, foi previsto um treinamento com apresentação do protocolo e sensibilização da equipe ao uso deste. Além disso, o protocolo também pode ser divulgado na intranet, revistas científicas e eventos na área da saúde.

#### Etapa III - Avaliação da qualidade por juízes

O instrumento de coleta de dados foi formado por duas partes: dados sociodemográficos e profissionais e o instrumento para avaliação do protocolo (AGREE II). O primeiro instrumento de caracterização dos juízes apresentava as variáveis sobre sexo, idade, estado de procedência, área de formação, tempo de formação, titulação, tempo de experiência com insulinoterapia em crianças internadas.

O AGREE II possui 23 itens, distribuídos em seis domínios: escopo e finalidade; envolvimento das partes interessadas; rigor do desenvolvimento; clareza da apresentação; aplicabilidade; e independência editorial^(15)^.

A avaliação global requer que o usuário do AGREE II faça um julgamento sobre a qualidade da diretriz, levando em conta os itens de avaliação considerados no processo. Ocorre após a avaliação dos 21 itens e é proposta pela escala Likert, variando entre 1 e 7, com a nota 1 significando qualidade mais baixa possível e a nota 7 a qualidade mais alta possível. Após essa avaliação, deve haver um parecer de recomendação da diretriz para ser utilizada na prática clínica^(15)^.

O AGREE II não define ponto de corte para classificar a qualidade. Essa decisão deve ser tomada pelo autor e orientada pelo contexto do uso do protocolo^(15)^. Ao final da avaliação, os juízes classificaram e avaliaram o protocolo da seguinte forma: recomendaria; recomendaria com limitações; e não recomendaria.

## RESULTADOS

Essa tecnologia foi intitulada “Protocolo de cuidados para uso seguro de insulina em crianças e adolescentes hospitalizados”. Na primeira etapa, foram identificados 1.760 artigos nas bases de dados, sendo excluídos 489 por serem duplicados. Foram elegíveis 1.271 para leitura de título e resumo. Após exclusão, 25 foram avaliados na íntegra, e 13 não responderam à questão de pesquisa. Logo, 12 artigos foram selecionados. Contudo, após leitura aprofundada e análise das referências, conseguiu-se recuperar quatro artigos, totalizando 16 artigos.

Os estudos foram divididos em categorias: técnica adequada no preparo da injeção subcutânea de insulina (sete artigos); uso da técnica da dobra cutânea (oito artigos); inspeção da pele e escolha do local de injeção (cinco artigos); uso de dispositivos adequados (dez artigos); infusão intravenosa de insulina (cinco artigos); prevenção de complicações e manejo da dor durante injeção (12 artigos); monitoramento de glicose sanguínea e padrões de hipoglicemia / hiperglicemia hospitalar (quatro artigos); armazenamento e conservação da insulina (sete artigos); e descarte correto dos perfurocortantes (sete artigos). Essas categorias determinaram a estrutura do protocolo em nove tópicos com a mesma denominação.

Cada tópico tem informações sobre objetivo, descrição do cuidado e observações as quais foram organizadas segundo evidências cientificas encontradas na revisão de escopo. Optou-se por destacar o título de cada tópico na cor laranja, que está relacionada à segurança do paciente, para melhor organização e visibilidade.

Participaram do processo de validação de conteúdo sete juízes, todas mulheres e enfermeiras, com média de 44+7,9 anos de idade e maioria procedente do estado de São Paulo (5), seguido do Ceará (2). Todos são especialistas, quatro mestres e uma doutora, e possuem média de 11 +8,4 anos de tempo de experiência clínica com crianças e adolescentes hospitalizados em uso de insulina.

No processo de avaliação, as pontuações foram alcançadas de acordo com cada domínio do instrumento (Quadro 1).

**Quadro 1:** Distribuição dos escores e adequabilidade do protocolo conforme os domínios do Appraisal of Guidelines for Research & Evaluation II, Fortaleza, Ceará, Brasil, 2024

**Domínio 1 – Escopo e finalidade**	**J1**	**J2**	**J3**	**J4**	**J5**	**J6**	**J7**	**TOTAL**
1. Os objetivos gerais da diretriz encontram-se especificamente descritos.	7	7	7	7	6	6	7	47
2. As questões de saúde cobertas pela diretriz encontram-se especificamente descritas.	7	7	7	7	5	6	7	46
3. A população (pacientes, público, etc.) a quem a diretriz se destina encontra-se especificamente descrita.	7	7	7	7	6	6	7	47
Total	21	21	21	21	17	18	21	140
Adequabilidade para o domínio 1 – 94,4%								
**Domínio 2 – Envolvimento das partes interessadas**	**J1**	**J2**	**J3**	**J4**	**J65**	**J6**	**J7**	**TOTAL**
4. A equipe de desenvolvimento da diretriz inclui indivíduos de todos os grupos profissionais relevantes.	7	7	7	7	5	7	6	46
5. Procurou-se conhecer as opiniões e preferências da população-alvo (pacientes, público, etc.).	7	1	7	7	7	6	6	41
6. Os usuários-alvo (pacientes, público, etc.) da diretriz estão claramente definidos.	7	7	7	7	7	6	7	48
Total	21	21	21	21	19	19	19	135
Adequabilidade para o domínio 2 – 90,4%								
**Domínio 3 – Rigor do desenvolvimento**	**J1**	**J2**	**J3**	**J4**	**J5**	**J6**	**J7**	**TOTAL**
7. Foram utilizados métodos sistemáticos para a busca de evidências.	7	7	7	7	7	6	7	48
8. Os critérios para a seleção de evidências estão claramente descritos.	7	7	7	7	7	6	7	48
9. Os pontos fortes e limitações do corpo de evidências estão claramente descritos.	7	7	7	7	6	6	7	47
10. Os métodos para a formulação das recomendações estão claramente descritos.	7	7	7	7	5	6	7	46
11. Os benefícios, efeitos colaterais e riscos à saúde foram considerados na formulação das recomendações.	7	7	7	7	6	6	7	47
12. Existe uma relação explícita entre as recomendações e as evidências que lhe dão suporte.	7	7	7	7	6	6	7	47
13. A diretriz foi revisada externamente por *experts* antes da sua publicação.	7	7	7	7	7	6	7	48
14. Um procedimento para atualização da diretriz está disponível.	7	7	7	7	7	6	7	48
Total	56	56	56	56	51	48	56	379
Adequabilidade para o domínio 3 – 96,1%								
**Domínio 4 – Clareza da apresentação**	**J1**	**J2**	**J3**	**J4**	**J5**	**J6**	**J7**	**TOTAL**
15. As recomendações são específicas e sem ambiguidade.	7	7	7	7	6	6	7	47
16. As diferentes opções de abordagem da condição ou problema de saúde estão claramente apresentadas.	7	7	7	7	6	6	7	47
17. As recomendações-chave estão facilmente identificadas.	7	7	6	7	7	6	7	47
Total	21	21	20	21	19	18	21	141
Adequabilidade para o domínio 4 – 95,2%								
**Domínio 5 – Aplicabilidade**	**J1**	**J2**	**J3**	**J4**	**J5**	**J6**	**J7**	**TOTAL**
18. A diretriz descreve os fatores facilitadores e as barreiras para sua aplicação.	7	7	7	7	6	6	7	47
19. A diretriz traz aconselhamento e/ou ferramentas sobre como as recomendações podem ser colocadas em prática.	7	7	7	7	6	6	7	47
20. Foram consideradas as potenciais implicações quanto aos recursos decorrentes da aplicação das recomendações.	7	7	7	7	6	6	6	46
21. A diretriz apresenta critérios para seu monitoramento e/ou auditoria.	7	4	7	7	6	6	7	44
Total	28	25	28	28	24	24	27	184
Adequabilidade para o domínio 5 – 92,8%								
**Domínio 6 – Independência editorial**	**J1**	**J2**	**J3**	**J4**	**J5**	**J6**	**J7**	**TOTAL**
22. O parecer do órgão financiador não exerceu influência sobre o conteúdo da diretriz.	7	7	7	7	7	6	7	48
23. Foram registrados e abordados os conflitos de interesse dos membros da equipe que desenvolveram a diretriz.	7	7	7	7	7	6	7	48
Total	14	14	14	14	14	12	14	96
Adequabilidade para o domínio 6 – 97,6%								

Observa-se que o domínio 3 (rigor do desenvolvimento) e o domínio 4 (clareza da apresentação) são os que obtiveram uma melhor pontuação. Já o domínio 2 (envolvimento das partes interessadas) obteve menor pontuação. Contudo, pode-se observar que todos os domínios superaram o valor mínimo de 80% definido com “qualidade satisfatória” e alcançaram índices superiores a 90%.

Quanto à avaliação global do protocolo e à recomendação de acordo com os juízes, destacamos que quatro atribuíram nota 7 e, três, nota 6, sendo a porcentagem total de avaliação global do protocolo igual a 92,8%. Considera-se, portanto, que o protocolo apresenta alta qualidade. Ademais, seis juízes recomendaram sem restrição o uso do protocolo, e apenas um recomendou com modificações.

Os juízes teceram sugestões acerca do conteúdo do instrumento para os itens e domínios do protocolo, conforme o AGREE II (Quadro 2), as quais, em sua maioria, foram acatadas.

**Quadro 2:** Sugestões e comentários dos juízes quanto ao conteúdo do protocolo, Fortaleza, Ceará, Brasil, 2023

Juiz	Sugestão	Tomada de decisão
Juiz 1	Descrever melhor os locais de aplicação de insulina com ilustrações.	Acatada e incluída ilustração com locais de aplicação.
Juiz 2	Acredito que um indicador interessante para o monitoramento/auditoria do protocolo pós-implementação seja a presença de complicações relacionadas à técnica de aplicação de insulina.	Acatada.
Juiz 3	Sugiro modificação do tópico que consta a técnica adequada de administração de insulina subcutânea: em ambiente hospitalar, a antissepsia da pele é feita em movimento unilateral com dois swabs de álcool 70% ou algodão embebido em álcool a 70%. Movimento circular não é utilizado mais.	Tópico III, em descrição do cuidado Mudar para: em ambiente hospitalar, a antissepsia da pele deve ser feita com movimentos unilaterais com swabs de álcool 70% ou algodão embebido em álcool a 70%.
Juiz 4	1. Incluir a ordem de injeção do ar junto com a observação de aspiração das insulinas NPH e regular para facilitar o manejo; 2. Fiquei na dúvida da frase que diz na observação que caneta é mais seguro para o profissional de saúde, uma vez que a maior parte dos locais não utiliza a agulha de segurança para caneta, sendo necessário desconectar, o que gera mais risco que a seringa; 3. Reavaliaria a frase a seguir no texto, colocaria talvez na observação: ângulo de 45 graus na agulha de 6 mm e a ordem de absorção.	Acatada.
Juiz 5	Sugiro incluir mais detalhadamente o passo a passo para administração de insulinas em canetas, bem como incluir o uso de tecnologias relacionadas às práticas seguras em insulinoterapia (i-port, CGM, bomba de insulina, etc.).	Acatada a sugestão de incluir o passo a passo para administração de insulinas em canetas. Porém, não foi possível incluir as tecnologias por não ser o foco do protocolo.
Juiz 7	Em relação a esse tópico (público-alvo), acredito que seria benéfico ao profissional de psicologia envolvido. Tratando-se de crianças, o desafio de garantir aceitação pra técnica e rodízio adequado.	Como trata-se de um protocolo para preparo e administração de insulina, acredita-se que, por ser um procedimento técnico, o psicólogo não se beneficiaria desse protocolo.

Ademais, além de sugestões, os juízes também teceram outros comentários e elogios, conforme falas a seguir:

*Parabéns pelo trabalho!* (J1)

*Gostei muito do trabalho realizado, muito bem escrito, de fácil compreensão e de grande valor para a prática clínica!!!* (J2)

*Parabéns pelo trabalho desenvolvido.* (J5)

*É necessária uma estratégia de treinamento muito dinâmica para atingir todos esses pontos; é um treinamento longo, sugiro fracionar em categorias com duração máxima de 30 min para melhor absorção e entendimento do time. Parabéns pelo projeto.* (J7)

## DISCUSSÃO

A etapa de construção dessa tecnologia, com conteúdo mapeado e sintetizado a partir da revisão, sustentou a parte teórica, abordando as principais práticas para o uso seguro e eficaz de insulina em crianças e adolescentes hospitalizados. O protocolo construído tem como objetivo oferecer recomendações baseadas em evidências científicas aos profissionais de saúde, em especial à equipe de enfermagem, que atua na assistência às crianças e adolescentes hospitalizados em uso de terapia insulínica.

O tratamento com insulina é um processo complexo, e a necessidade desse hormônio em crianças e adolescentes é dinâmica, devido às mudanças fisiológicas e comportamentais características dessa faixa etária, bem como aos padrões de rotinas diárias, presença de doenças concomitantes, entre outros fatores^(16)^. Nesse sentido, as tecnologias, quando aplicadas e testadas no ambiente hospitalar, apresentam resultados satisfatórios nos processos assistenciais, visto que facilitam a dinâmica do serviço dos profissionais, reduzem a possibilidade de eventos adversos oriundos da assistência prestada e contribuem para a segurança do paciente^(17)^.

O estudo realizado por Poppy *et al.* ^(18)^ mostrou que a taxa de eventos hipoglicêmicos em crianças hospitalizadas em uso de insulina reduziu de 1,4 eventos evitáveis, por 100 dias de insulina, para 0,4, uma redução de 28% após a unidade hospitalar estabelecer múltiplas intervenções, como desenvolvimento de protocolos, educação permanente dos profissionais sobre a insulinoterapia e vigilância em tempo real dos pacientes em risco de hipoglicemia inesperada.

O cuidado prestado à criança em processo de hospitalização se torna cada vez mais aprimorado, pois a busca por melhorias nos instrumentos utilizados no cotidiano assistencial, fundamentados cientificamente a partir de métodos que os tornem válidos, vem sendo ponto crucial desenvolvido por enfermeiros que atuam, com o intuito de transformar a prática diante de inovações metodológicas^(19)^.

Tecnologias de cuidados elaboradas a partir de evidências robustas e avaliadas por especialistas na temática contribuem positivamente com a assistência, tendo em vista que aliam ciência e a experiência cotidiana de profissionais^(20)^.

Outros estudos relacionados à temática do presente estudo também têm utilizado o AGREE II, como o realizado na Palestina, que visava à avaliação de uma diretriz clínica para diabetes mellitus. E apresentou índices inferiores aos encontrados em nosso estudo, com uma avaliação de qualidade baixa: domínio 1 (66,7%); domínio 2 (35%); domínio 3 (36,5%); domínio 4 (61,5%); domínio 5 (27%) e domínio 6 (40%). A utilização foi recomendada com modificações por 12 avaliadores e não recomendada por quatro avaliadores^(21)^.

Algumas orientações para as práticas seguras relacionadas ao uso de insulina em crianças e adolescentes internados são: a dobra cutânea é obrigatória em crianças menores de 6 anos e em adolescentes magros; as seringas de agulha fixadas permanentemente oferecem melhor precisão da dose e espaço morto reduzido; as agulhas curtas (4, 5 e 6 mm de comprimento) são ideais para todos os pacientes, e seu uso é seguro para qualquer indivíduo; a absorção de insulina injetada em locais com lipo-hipertrofia é imprevisível, podendo haver maior variabilidade glicêmica^(22)^.

Alguns cuidados devem ser realizados no preparo da insulina: insulina turva ou pré-misturada deve ser ressuspensa antes de cada injeção; uma injeção de ar de duas unidades deve ser feita antes de preparar a dose pretendida para garantir fluxo livre e desobstruído na agulha da caneta^(23)^.

A insulina deve ser administrada no tecido subcutâneo para garantir a absorção ideal. É importante inspecionar a pele antes e depois da injeção, inserir a agulha corretamente no tecido subcutâneo, deixar a agulha dentro do tecido o tempo suficiente para toda dose de insulina ser absorvida e realizar rodízio dos locais de aplicação. Essas ações contribuem para um controle metabólico eficiente^(24)^.

O uso de agulhas curtas e finas (4 mm e 5 mm) na administração de insulina melhora a técnica de injeção ao diminuir a dor durante o processo de injeção, reduzir a chance de injeção intramuscular e melhorar a taxa e a integralidade da absorção de insulina^(25)^.

A injeção usando frasco e seringa é considerada um meio eficaz e mais barato de administração de insulina. Contudo, apresenta algumas inconveniências, como baixa precisão da dose, falta de aceitação social, medo, dificuldade de transporte. Já as canetas de insulina, comparadas com as seringas, proporcionam mais flexibilidade, precisão e discrição, contribuindo para melhorar a adesão ao tratamento^(26)^.

O armazenamento e a conservação corretos são imprescindíveis para garantir a eficácia da insulina e sucesso na terapia. A insulina em uso pode ser mantida em temperatura ambiente (abaixo de 30°C) por até 30 dias. Ressalta-se que administrar insulina refrigerada pode ser desconfortável para o paciente^(23)^.

Por fim, o descarte seguro de objetos cortantes usados deve ser praticado em ambientes domésticos e hospitalares para evitar ferimentos por agulhas relacionados a agulhas de insulina^(27)^.

Complicações locais da terapia com insulina são comuns em pacientes com diabetes, como infecções e lipodistrofia, sendo essa última a de maior impacto no controle metabólico. Estudo realizado na Turquia em crianças com diabetes tipo 1 encontrou essa complicação em 11,8% dos pacientes. Os pacientes com lipodistrofia utilizavam doses medianas mais altas de insulina (0,97 U/kg/dia) do que aqueles que não usavam (0,78 U/kg/dia; *p =* 0,016), e apresentavam hipoglicemia frequente (*p* = 0,007)^(28)^.

O rodízio incorreto dos locais de administração de insulina e a reutilização da agulha por mais de cinco vezes estão significativamente associados ao desenvolvimento de lipodistrofia, destacando assim a importância da técnica correta de injeção^(29)^.

Em indivíduos hospitalizados com diabetes, é necessário monitorar a glicemia conforme seu quadro clínico. Para aqueles que estão se alimentando, o monitoramento deve ser realizado antes das refeições; para os que estão em dieta zero, recomenda-se verificar a glicemia capilar a cada quatro a seis horas. O monitoramento mais frequente da glicemia (30 minutos a 2 horas) é necessário para o uso seguro da terapia intravenosa com insulina. Destaca-se que, se o resultado da glicose não se correlacionar com o estado clínico do paciente, deve ser ratificado pela medição de uma amostra laboratorial^(30)^.

Ratifica-se que a eficácia da terapia com insulina depende do uso correto da técnica de injeção. No entanto, apesar das evidências de que a técnica eficaz é essencial para minimizar os erros e melhorar o controle glicêmico, ainda há necessidade de sensibilização entre os pacientes e profissionais de saúde sobre a importância da otimização das técnicas de injeção de insulina^(25)^.

A implementação de programas educativos de administração de insulina em unidades de saúde, como hospitais, enfermarias e Unidades de Terapia Intensiva, é essencial para garantir o uso seguro e eficaz da insulina, estabelecendo procedimentos padrão, reduzindo o risco de prescrição e administração inadequadas de insulina e melhorando os resultados dos pacientes^(25,26,27)^.

Esses programas devem abordar todos os aspectos do uso seguro e racional da insulina, além de indicadores que podem ser usados para quantificar o sucesso do programa, tais como redução de erros na administração de insulina, redução nas taxas de hipoglicemia, porcentagem de manejo correto da hipoglicemia, porcentagem de pacientes e profissionais que concluíram o programa sobre insulina, descarte correto de agulhas e seringas^(27)^, indicadores semelhantes aos do protocolo.

### Limitações do estudo

As limitações do estudo foram determinadas pela incipiência de estudos com altos níveis de evidências que elucidem as boas práticas específicas sobre uso de insulina voltadas para o público pediátrico. Acredita-se que futuras pesquisas nessa temática são essenciais para aprimorar conhecimentos e garantir uma atenção qualificada e segura.

### Contribuições para a área da enfermagem

Esse protocolo apresenta orientações seguras para a prática da insulinoterapia em crianças e adolescentes hospitalizados, contribuindo significativamente para o aprimoramento da prática clínica de enfermagem, promovendo a qualidade do cuidado.

O uso desse protocolo favorecerá a padronização da insulinoterapia, com redução dos erros de medicação associados à insulina, especialmente no âmbito da pediatria, já que possui particularidades como dosagens e reações adversas, fato que poderá contribuir para melhores resultados clínicos e metabólicos de crianças e adolescentes.

## CONSIDERAÇÕES FINAIS

O “Protocolo de cuidados para uso seguro de insulina em crianças e adolescentes hospitalizados” foi construído com rigor metodológico, avaliado e recomendado por juízes especialistas para uso por profissionais de saúde no contexto da insulinoterapia na pediatria.

O produto desta pesquisa poderá contribuir para a prática clínica da enfermagem, de modo a agregar conhecimentos, normatizando condutas e mitigando erros e/ou complicações relacionados ao uso de insulina em crianças e adolescentes hospitalizados.

Sugere-se a realização de estudos posteriores para a implementação e monitoramento, assim como para avaliar o impacto do uso do protocolo na qualidade e segurança do cuidado à criança e adolescente em insulinoterapia no âmbito hospitalar, além da atualização periódica do protocolo.

## Data Availability

https://doi.org/10.17605/OSF.IO/JN6CQ
